# Adult-Onset Still’s Disease With Suspected Steroid Hypersensitivity: A Case Report

**DOI:** 10.7759/cureus.91112

**Published:** 2025-08-27

**Authors:** Aaliya Abdul Gafoor, Abdullah Khalid, Tariq Karim

**Affiliations:** 1 Internal Medicine, Jabal Sina Medical Centre, Ajman, ARE; 2 General Practice, Jabal Sina Medical Centre, Ajman, ARE

**Keywords:** adult onset stills disease, fever of unknown origin, hyperferritin, rash, steroid hypersensitivity

## Abstract

Adult-onset Still’s disease (AOSD) is a rare, systemic auto-inflammatory disorder characterised by quotidian spiking fevers, arthralgia or arthritis, and a salmon-pink maculopapular rash. Corticosteroids are the mainstay of treatment, but rarely, hypersensitivity reactions to steroids can complicate management.

Here, we report a complicated case of a 39-year-old woman who presented with recurrent high-grade fever, arthralgia, rash, sore throat, and lymphadenopathy. Laboratory investigations revealed elevated inflammatory markers and serum ferritin. She met Yamaguchi criteria for AOSD and responded well to intravenous methylprednisolone, but later developed a pruritic rash suggestive of a hypersensitivity reaction. Her treatment was switched to oral prednisolone, after which her symptoms resolved and she continued to improve.

This case highlights the diagnostic challenges of AOSD and the need for awareness of rare steroid hypersensitivity reactions. In patients with suspected AOSD who develop new or atypical cutaneous symptoms during steroid therapy, clinicians should consider the possibility of hypersensitivity. Early recognition and management can help achieve favourable outcomes in complex cases.

## Introduction

Adult-onset Still’s disease (AOSD) is an uncommon, systemic auto-inflammatory disorder characterised by quotidian spiking fevers, arthritis or arthralgia, and a salmon-pink maculopapular rash. It was first described by Bywaters in 1971 and is often challenging to diagnose due to its non-specific clinical presentation and overlap with infections, autoimmune diseases, and malignancies [1-3].

At least seven sets of diagnostic criteria have been proposed for AOSD, with the Yamaguchi criteria being the most widely used due to their high sensitivity [4]. Diagnosis is largely clinical, supported by laboratory markers of systemic inflammation, and requires exclusion of similar conditions.

Treatment typically begins with non-steroidal anti-inflammatory drugs (NSAIDs) and corticosteroids. Corticosteroids are considered the mainstay of therapy and usually result in rapid clinical improvement. In cases of relapse or resistance, disease-modifying anti-rheumatic drugs (DMARDs) and biological agents are introduced [1,3].

Although corticosteroids are widely regarded as safe and effective, hypersensitivity reactions such as urticaria, angioedema, or even anaphylaxis can occur. These reactions are particularly concerning in diseases like AOSD, where corticosteroids play a central role in disease control. Such hypersensitivity poses a significant therapeutic dilemma, requiring alternative strategies such as early use of steroid-sparing agents, careful desensitisation protocols, or individualised biologic therapy [1].

In this report, we present a diagnostically and therapeutically challenging case of AOSD in a middle-aged woman, where the clinical course was complicated by a suspected hypersensitivity reaction to corticosteroids. This case highlights the importance of vigilance for atypical drug reactions and emphasises the need for individualised management in complex auto-inflammatory conditions [4,5].

## Case presentation

Initial presentation

A 39-year-old woman with no known co-morbidities presented to the Family Medicine outpatient department with high-grade fever for the past three days. She reported temperatures up to 40°C, associated with chills, sore throat, nasal congestion, and generalised body and joint pain. There was no relevant medical or family history and no known allergies. She also reported recent exposure to a pet cat.

On examination, she was febrile with an erythematous pharynx. No rash or lymphadenopathy was noted, and systemic examination was otherwise unremarkable. An initial diagnosis of acute pharyngitis with allergic urticaria was made. She was discharged with oral medications, including azithromycin, an antipyretic, and an antihistamine. 

Second presentation (day 2)

Two days later, she returned with persistent fever (around 39°C) and a salmon-pink maculopapular rash that was non-pruritic. The rash appeared in patches over both upper limbs, legs, back, and abdomen without complete coverage. Given the recent introduction of an antibiotic and her pet cat exposure, a drug reaction or allergy was initially suspected.

Lab investigations showed elevated C-reactive protein (CRP) and low haemoglobin (Hb). She was given a short course of oral prednisolone, and intravenous ceftriaxone was administered for three days. Her fever and rash subsided, and she showed significant improvement with treatment.

Third presentation (day 10)

On day 10, she presented again with generalised body aches, arthralgia, facial puffiness, and mild abdominal discomfort. A broader differential was considered. Investigations included CBC, liver and renal function tests, and urine analysis. The only significant finding was a raised WBC count; other parameters were within normal limits.

Although she was afebrile at this visit, empirical antimicrobial therapy with intravenous ceftriaxone (2 g for three days) followed by oral cefuroxime (500 mg twice daily) was commenced in view of leukocytosis and her recent febrile illness. In addition, a short course of oral prednisolone (20 mg twice daily) was prescribed for her persistent arthralgia and systemic symptoms.

Fourth presentation (day 13)

Three days later, she re-presented with high-grade fever, chills, myalgia, arthralgia, and cervical lymphadenopathy. She was referred to the Internal Medicine Consultant for further evaluation.

A comprehensive workup was undertaken, including chest X-ray, peripheral smear for malarial parasites, Widal test, blood and throat cultures, serum ferritin, erythrocyte sedimentation rate (ESR), and rheumatoid factor. The chest X-ray was unremarkable, and the malarial smear was negative. Laboratory results showed raised WBC count, CRP, ESR, and serum ferritin. Both Widal and rheumatoid factor tests were negative, and no organisms were isolated from blood or throat cultures.

Differential diagnosis

In a patient presenting with fever, salmon-pink maculopapular rash, and lymphadenopathy, the differential diagnosis included infections, other autoimmune diseases, malignancy, and drug reactions. However, a negative malarial smear, sterile blood and throat cultures, negative Widal test, negative rheumatoid factor, normal renal and liver function, and the absence of cytopenias made infection, malignancy, and other autoimmune conditions less likely.

Drug reactions were also considered; however, recurrence of symptoms in the absence of any recent drug exposure, together with elevated inflammatory markers, fulfilment of Yamaguchi criteria, and raised ferritin, supported AOSD as the more consistent diagnosis.

Provisional diagnosis and treatment

A provisional diagnosis of adult-onset Still’s disease was made, and the patient was started on intravenous methylprednisolone. She demonstrated rapid clinical improvement. Within 48 hours, her fever subsided, rash resolved, and repeated labs showed a decrease in CRP to 0.57 mg/L, ESR to 50 mm/hr, and WBC to 13 ×10⁹/L. Laboratory findings from each visit are presented in Table 1. Based on the clinical criteria and significant response to corticosteroids, the diagnosis of AOSD was confirmed according to Yamaguchi’s criteria.

**Table 1:** Laboratory results CRP: C-Reactive Protein; WBC: White Blood Cells; PLT: Platelets; ALT: Alanine Aminotransferase; AST: Aspartate Aminotransferase; ESR: Erythrocyte Sedimentation Rate; RF: Rheumatoid Factor; Blood C/S: Blood Culture and Sensitivity

Parameters	05 Jun (Day 2)	13 Jun (Day 10)	16 Jun (Day 13)	17 Jun	19 Jun	22 Jun	25 Jun	Reference range
CRP	85.42	-	84.28	-	26.28	0.57	-	< 5.0 (mg/L)
Haemoglobin	11.4	11.3	11.3	-	9.5	10.8	-	12.0-15.0 (g/dl)
WBC	10	13.28	17.03	-	19.15	13.13	-	4.00-10.00 (10^3/Ul)
PLT	266	341	240	-	266	299	-	150-410 (10^3/Ul)
HbA1c	-	6.18	-	-	-	6.67	-	Non-Diabetic: < 5.7 Prediabetic: 5.7-6.4 Diabetic: > 6.5 %
Vitamin D	-	16.87	-	-	-	16.81	-	Deficient: <20 Insufficient: 20-30 Sufficient: >30 (ng/mL)
Creatinine	-	0.64	-	-	-	0.62	-	0.50-0.90 (mg/dL)
Urea	-	26.51	-	-	-	28.33	-	17.0-43.0 (mg/dL)
Uric Acid	-	5.06	-	-	-	4.55	-	2.30-6.10 (mg/dL)
ALT	-	21.11	-	-	-	24	-	<34 (U/L)
AST	-	18.7	-	-	-	10.23	-	<31 (U/L)
Total bilirubin	-	0.36	-	-	-	0.33	-	0.30-1.10 (mg/dL)
Total protein	-	6.89	-	-	-	6.38	-	6.6-8.3 (g/dL)
Albumin	-	4.09	-	-	-	4.06	-	3.50-5.30 (g/dL)
Iron	-	59.73	-	-	-	28.68	-	37-145 (ug/dL)
Widal test	-	-	Negative	-	-	-	-	-
ESR	-	-	70	-	50	-	-	0-20 (mm/hr)
Ferritin	-	-	-	407.4	-	-	-	12-135 (ng/dL)
RF	-	-	-	negative	-	-	-	-
Throat Culture	-	-	-	-	No growth	-	-	-
Blood C/S	-	-	-	-	-	-	No growth	-
Urine analysis	-	Normal	-	-	-	-	Normal	-

Steroid hypersensitivity

Although the patient initially responded well to intravenous methylprednisolone, she later developed a recurrence of the rash, which was now pruritic. It presented as a persistent erythematous maculopapular rash, predominantly affecting the upper limbs and abdomen. The clinical features were more suggestive of a drug-induced hypersensitivity reaction, as pruritic rashes are not typically associated with AOSD. This was most consistent with a Type IV (delayed, T-cell-mediated) hypersensitivity reaction manifesting as a maculopapular (morbilliform) drug eruption. A hypersensitivity reaction to methylprednisolone was therefore suspected.

Outcome

Her treatment was switched to oral prednisolone, after which the rash promptly resolved. She was discharged on oral corticosteroids with instructions for close outpatient follow-up. She continued to report sustained improvement in symptoms during follow-up visits, supporting a favourable response to therapy.

## Discussion

AOSD is a rare, multisystem inflammatory disorder of unknown etiology marked by quotidian spiking fevers, salmon-pink maculopapular rash, inflammatory polyarthritis and systemic features such as lymphadenopathy and hepatosplenomegaly. It has an estimated annual incidence of 0.1-0.4 cases per 100,000 population in Europe, shows a slight female predominance, and follows a bimodal age distribution with peaks between 15-25 years and 36-46 years. Although “Still’s disease” is more commonly seen in children, where it is referred to as systemic juvenile idiopathic arthritis, it can also affect those over the age of 16, in which case it is termed AOSD, a much rarer presentation [6].

Although the exact cause remains unknown, several theories have been proposed regarding its etiology, including genetic susceptibility and environmental factors. The prevailing hypothesis suggests that AOSD is a reactive syndrome in which certain infections act as a trigger for AOSD in those with a genetic predisposition. Infections such as Yersinia enterocolitica and Mycoplasma pneumoniae have been linked in some cases and studies have shown associations with specific human leukocyte antigen (HLA) types like B17, B18, and DR2. There have even been reports of AOSD occurring in twins, suggesting a possible genetic component [6]. 

Pathophysiologically, it is driven by a dysregulation of the innate immune system with elevated levels of interleukin (IL)-1, IL-6, IL-18, and tumour necrosis factor alpha (TNF-α) playing a central role in this [6]. Clinical patterns can vary, with some patients having a single flare (monophasic), while others experience repeated episodes (intermittent or polycyclic). Diagnosis is usually made using the Yamaguchi criteria (Table 2), which are considered the most sensitive. 

**Table 2:** Yamaguchi Criteria A diagnosis requires the presence of five features, with at least two being major diagnostic criteria [4]. ALT: Alanine Aminotransferase; AST: Aspartate Aminotransferase; LDH: Lactate Dehydrogenase

Yamaguchi Criteria	
Major Criteria:	Fever ≥ 39°C lasting ≥ 1 week.
	Arthralgia or arthritis lasting ≥ 2 weeks
	Typical skin rash: maculopapular, non-pruritic, salmon-pink rash during febrile episodes
	Leukocytosis ≥ 10,000/ml with ≥ 80% granulocytes
Minor Criteria:	Sore throat
	Lymphadenopathy
	Hepatomegaly or splenomegaly
	Abnormal liver function studies (↑ AST, ALT, LDH)
	Negative anti-nuclear antibody and rheumatoid factor
Exclusion Criteria:	Infection
	Malignancy
	Other rheumatic diseases

Our patient met seven criteria, four of which were major and three minor. The major criteria included a fever of 39°C, arthralgia, salmon-pink maculopapular rash, and leukocytosis. The minor criteria were sore throat, lymphadenopathy, and negative rheumatoid factor.

The differential diagnosis includes infection, autoimmune disease, malignancy, and drug-induced reactions. Infectious causes such as malaria and typhoid were excluded through a negative peripheral smear, negative Widal test, and sterile blood and throat cultures. Malignancy was considered, but no cytopenias or organomegaly were present, and liver and renal function remained normal. Autoimmune conditions such as vasculitis and connective tissue disease were also considered, though rheumatoid factor was negative. Drug reactions, including antibiotic-related exanthema, were initially considered given her prior medication exposure. However, the recurrence of symptoms in the absence of these drugs, together with elevated inflammatory markers, features of Yamaguchi criteria, and raised ferritin, supported AOSD as the more consistent diagnosis.

Another important finding was elevated ferritin. Although not specific to AOSD, high ferritin levels, when combined with clinical and lab findings, can strongly suggest the diagnosis. In AOSD, ferritin is usually five times above the upper limit of normal or even higher. Serial serum ferritin levels are also helpful in monitoring treatment response [7].

AOSD is initially treated with non-steroidal anti-inflammatory drugs and corticosteroids as the first line (medium-high to high doses (0.5-1 mg/kg/day) of prednisone), and for those who fail to achieve full remission on corticosteroids, DMARDs or biological agents are introduced. In our case, the patient received earlier short courses of oral prednisolone, which did not achieve sustained remission, likely because the doses and duration were insufficient to control active disease. She was later started on intravenous methylprednisolone, which produced rapid clinical improvement, but subsequently developed a worsening, pruritic rash suggestive of a hypersensitivity reaction. Although rare, allergic reactions to methylprednisolone have been reported and may be caused by the steroid itself or by the preservatives, adjuvants, or stabilisers used in its formulation. In most cases, it is the succinate ester of hydrocortisone that causes type I hypersensitivity reactions in systemic corticosteroids [8]. This was likely the case here as she tolerated oral prednisone well and showed immediate improvement thereafter.

This case highlights the importance of following up with patients when there is diagnostic uncertainty. The stepwise progression of symptoms, including persistent fever, arthralgia, salmon-pink maculopapular rash, and later cervical lymphadenopathy, suggested something more concerning than a simple viral infection or allergy. Failure to recognise these clinical patterns early can lead to delayed diagnosis and unnecessary treatment before AOSD is considered. The case was further complicated by steroid hypersensitivity, which introduced symptoms that could easily be mistaken for the disease itself. This emphasises the need to approach each visit with fresh clinical judgment as even subtle details, such as the presence of pruritus, may help differentiate between active disease and an allergic reaction.

## Conclusions

AOSD should be considered as one of the differential diagnoses of pyrexia of unknown origin (PUO). It is essential to include serum ferritin testing as part of the initial workup to rule out more common causes of fever. Markedly elevated ferritin levels, often exceeding 3,000 ng/ml and occasionally rising above 10,000 ng/ml, can serve as a diagnostic clue along with Yagamuchi’s criteria. Our patient’s ferritin was only mildly high at 407 ng/mL; however, even a moderate elevation can be significant when interpreted in the context of other Yamaguchi criteria features.

This case also highlights the importance of ensuring adequate corticosteroid dosing, as short, low-dose courses may be insufficient to achieve disease control. Furthermore, clinicians should remain vigilant for hypersensitivity reactions to corticosteroids, which, although rare, can complicate management and mimic features of the underlying disease.
